# Family care during the first COVID-19 lockdown in Germany: longitudinal evidence on consequences for the well-being of caregivers

**DOI:** 10.1007/s10433-023-00761-2

**Published:** 2023-05-11

**Authors:** Katja Möhring, Sabine Zinn, Ulrike Ehrlich

**Affiliations:** 1 Faculty of Social Sciences, Economics and Business Administration, University of Bamberg, Bamberg, Germany; 2 Mannheim Centre for European Social Research (MZES), University of Mannheim, Mannheim, Germany; 3 Socio-Economic Panel (SOEP), German Institute for Economic Research (DIW Berlin), Berlin, Germany; 4 German Centre of Gerontology (DZA), Berlin, Germany; 5 Departement of Social Sciences, Humboldt University, Berlin, Germany

**Keywords:** Corona pandemic, Depression, First difference regression, Informal care, Life satisfaction, SOEP-CoV

## Abstract

We examine changes in the well-being of family caregivers during the early phase of the COVID-19 pandemic in Germany, using data from the German Socio-Economic Panel (SOEP) and the SOEP-CoV study. The COVID-19 pandemic posed an extraordinary challenge for family caregivers, as care recipients are a high-risk group requiring special protection, and professional care services were severely cut back. The specific situation of the COVID-19 pandemic allows us to re-examine the caregiver stress process model. Using first difference regression models, we analyse changes in general life satisfaction and depressive symptoms (PHQ-4 score) among family caregivers between 2019 and spring 2020, differentiating by care intensity and duration of the care episode. Caregivers show similar changes in well-being as non-caregivers: a simultaneous increase in depressive symptoms and life satisfaction between 2019 and 2020. However, our results reveal heterogeneity within the group of family caregivers as we find differences according to caregiving dynamics and intensity. Among the group of continuing caregivers, high-intensity caregivers experience a larger increase in life satisfaction, and low-intensity caregivers a smaller increase in life satisfaction, compared to non-caregivers. Our results therefore provide some support for the role enhancement hypothesis for continuing caregivers with high time commitment.

**Supplementary Information:**

The online version contains supplementary material available at 10.1007/s10433-023-00761-2.

## Introduction

Approximately 8 million people in Germany (11.5% of the adult population) provide help, support or care (‘family caregivers’ in the following) for a partner, parent or other family members (or sometimes neighbours or friends) suffering from poor health, disability or age-related frailty (Rothgang and Müller 2022). Unlike professional care providers, who are usually trained and employed for their care services, family caregivers are typically untrained and unpaid (Lilly et al. 2007; Van Houtven 2015). However, the German welfare system relies heavily on the care services provided by family caregivers; although a small number of people are cared for by professional care providers in nursing homes, most care-dependents receive care from family members or friends in their own home (Destatis 2020). However, only 31.5% of family caregivers reside with their care recipients (Ehrlich and Kelle 2019). While providing family care can be a rewarding task (e.g. Moen et al. 1995), research mainly supports the notion that family caregiving operates as a stressor and impacts negatively on the well-being of caregivers (e.g. Kaschowitz and Brandt 2017; Sacco et al. 2020).

The COVID-19 pandemic posed an extraordinary challenge to family caregivers. Care recipients, and most family caregivers themselves, were at an increased risk of severe sickness from COVID-19 due to their higher age, impaired health and/or existing chronic conditions (Fischer and Geyer 2020; Schilling et al. 2021). During the first wave of the pandemic, the death toll was especially high among the old (60–79 years) and very old (80 years and older) (Schilling et al. 2021). As testing possibilities were not widely available and a vaccine had not yet been developed, high insecurity existed in how to manage the situation and protect the elderly population. Common strategies included isolating the elderly, cutting professional care services, and suspending access to nursing homes for new patients (RKI, 2021; Rothgang et al. 2020). These measures constrained family caregivers’ formal and informal support networks. Building on the stress process/role strain framework (Moen et al. 1995; Pearlin et al. 1990), the spread of COVID-19 was an additional stressor in the relationship between family care and well-being, leading to an increase in the care burden, stress and emotional strain of family caregivers. However, according to the role enhancement theory (Moen et al. 1995), the COVID-19 pandemic could also positively affect the well-being of caregivers; feeling that they perform an even more crucial role for their care recipient and society may have contributed to increased self-esteem and a strengthened sense of identity.

There has been little research on whether and how the pandemic has affected the well-being of family caregivers using a longitudinal approach and detailed information on caregiving characteristics (for an exception, see Ehrlich et al. 2022). Therefore, our study addresses whether—and if so to what extent—the well-being of family caregivers was affected during the first wave of the COVID-19 pandemic, and what differences exist with regard to family care dynamics (new caregiver, continuing caregiver) and intensity (low- vs. high-intensity care). We investigate two well-being outcomes: general life satisfaction and depressive symptoms (PHQ-4 score).

In answering this research question, our study contributes to the literature in two major ways. First, it allows to re-examine whether the relative strengths of prominent theories in the area of family care and well-being also hold true during the COVID-19 outbreak and subsequent containment measures. Second, previous studies addressing the well-being of family caregivers during the COVID-19 pandemic have mainly used caregiver-specific, cross-sectional or non-probability data (e.g. Brandt et al. 2021; Budnick et al. 2021; Rodrigues et al. 2021), have not been able to detect causal relationships within a representative sample, have not focused on specific sub-groups of caregivers (Whitley et al. 2021 only include co-residing caregivers) or have not accounted for family care intensity (Ehrlich et al. 2022). In contrast, our study uses data from the German Socio-Economic Panel (SOEP) and its special Corona study, SOEP-CoV (2020, www.soep-cov.de), which took place during the first lockdown in spring 2020 and in the period immediately afterwards. The SOEP is a longitudinal household study relying on probability samples, which enables the examination of changes in the well-being of caregivers between 2019 and the first wave of the pandemic in 2020, differentiating along care dynamics and intensity.

## Theory and previous research

### Family caregiving and well-being

Family care is a broad concept which refers to both care provided in legally recognised relationships and care in other forms of relationships such as biological, step and adopted families, and families by choice. The most important attribute of family caregiving is not necessarily a familial relationship, but rather a familial commitment between caregiver and care recipient (Freedman and Wolff 2020). Fulfilling this commitment can occur at any point during the life course (Moen et al. 1994) and is expressed in the caregiver's attention to the care recipient’s social, psychological, emotional and physical needs (Knijn and Kremer 1997).

Two main scenarios of how family care might impact on individuals’ well-being have been proposed. According to the role strain perspective and the caregiver stress process model, family care provision leads to negative well-being outcomes as “[…] there is a fixed quantity of time, energy, and commitment available for role-related responsibilities [and the] […] role of caregiving is taken on in addition to ongoing family and nonfamily obligations, producing the very real possibility of overload and strain, and consequently psychological distress” (Moen et al. 1995, p. 260). The caregiver stress model predicts the same outcome, albeit through a slightly different mechanism; family care can transform “from the ordinary exchange of assistance among people standing in close relationship to one another to an extraordinary and unequally distributed burden” (Pearlin et al. 1990, p. 583). Family caregiving is “potentially a fertile ground for persistent stress” that negatively affects individuals’ well-being (Pearlin et al. 1990, p. 583). Stress and strain related to family care tasks may arise from ‘caring for’ the care recipient, but also from ‘caring about’ and observing a loved one suffering from poor health, disability or age-related frailty (Bobinac et al. 2010; Bom et al. 2019).

The role enhancement approach takes a contrasting perspective from the negative implications of caregiving proposed by the role strain persepctive and the stress process model. According to the role enhancement perspective, family caregiving can be perceived as rewarding, increasing the subjective well-being of family caregivers. As “[s]ocial integration, in the form of multiple roles, augments an individual’s power, prestige, resources, and emotional gratification, including social recognition and a heightened sense of identity”, the family caregiver role is associated with benefits to well-being (Moen et al. 1995, p. 260).

Previous longitudinal research analysing probability-based population data mainly supports the role strain and stress process perspectives; transitioning into family care is associated with increasing depressive symptoms (Coe and Van Houtven 2009; Hiel et al. 2015; Kaschowitz and Brandt 2017; Kaschowitz and Lazarevic 2020; Marks et al. 2002; Zwar et al. 2020) and decreasing quality of life (Rafnsson et al. 2017; Sacco et al. 2020) or life satisfaction (Gerlich and Wolbring 2021). However, there is also research showing a positive relationship, or no relationship at all (e.g. Hajek and König 2016; Leigh 2010). Furthermore, variation exists according to caregiving duration and intensity. While empirical studies show a decline in well-being after the *transition into* family care (e.g. Sacco et al. 2020; Zwar et al. 2020), they yield inconclusive answers on if and how the well-being of the caregiver may change over the longer course of the care episode. Although Lacey et al. (2019) and Sacco et al. (2020) found that continuing caregivers are worse-off in terms of well-being than non-caregivers, they did not observe an additional decline in well-being over time. However, Coe and Van Houtven (2009) have shown that continuing care is associated with losses in well-being. Furthermore, studies demonstrate that the negative effect of family care on well-being tends to be stronger for caregivers providing more hours of care (Chen et al. 2019; Hirst 2005; Sacco et al. 2020). In summary, previous research provides mixed results on the relationship between family caregiving and the well-being of caregivers. Therefore, heterogeneity in family caregiving needs to be considered, especially with respect to care intensity and dynamics.

### Family caregiving during the COVID-19 pandemic

A general lockdown with stay-at-home orders was in place in Germany starting from 22 March 2020, which was extended twice until the beginning of May (Naumann et al. 2020). This included a closure of schools, kindergartens, and almost all public and leisure facilities. People were told to stay at home but could leave the home for walks and outdoor activities. Meetings with others were only allowed outdoors and with no more than one member of another household (Bundesregierung 2020). The effects of the COVID-19 pandemic and the related containment measures on individuals’ well-being and mental health varied for different groups in society, depending not only on their current socio-economic and health status, but also on their coping strategies (Zacher and Rudolph 2021; Möhring et al. 2021). For family caregivers, it seems likely that the “stress process” and ﻿“role strain﻿” dominated during the first wave of the COVID-19 pandemic, rather than the role enhancement perspective. Family caregivers predominantly provide care for frail or elderly people at particularly high risk of severe disease in cases of COIVD-19 infection (especially at the beginning of the pandemic when vaccination was unavailable). Moreover, many family caregivers are elderly and thus also belonged to high-risk groups (Fischer and Geyer 2020). Thus, family caregivers faced increased stress during the first wave of the pandemic from protecting themselves and their care recipients from COVID-19 infection (Gilligan et al. 2020). At the same time, though social-distancing measures had eased by the end of the first pandemic wave, a cure or vaccine was still not available and family caregivers might still have felt the need to reduce social contacts.

Furthermore, the family care situation also changed during the COVID-19 pandemic; the availability of professional care services was restricted, leading to a shrinking of formal support possibilities for family caregivers. During the first COVID-19 lockdown, outpatient services were completely or partially cancelled for tasks that could be fully or partially taken on by relatives living in the household (Räker et al. 2021). In care homes, admission stops were widely implemented (Ott 2020; RKI 2021; Rothgang et al. 2020). As a result of social-distancing practices, family caregivers’ informal support networks were also impaired (Eggert et al. 2020). This likely led to more unmet support needs, more individual care hours and more stress (Gilligan et al. 2020; Raiber et al. 2022; Verbakel et al. 2018); about 25% of family caregivers in Germany expressed a need for more support during the COVID-19 pandemic (Klaus and Ehrlich 2021).

Empirical results from the UK support the notion of a lowering of the well-being of family caregivers during the Corona pandemic (Whitley et al. 2021 for caregivers who look after a household member), and the first empirical results for Germany show that the pandemic was associated with increased feelings of loneliness among continuing caregivers (Ehrlich et al. 2022). Taken together, these theoretical considerations and empirical results lead us to the formulation of our first hypothesis: *family caregivers will have experienced a larger decline in well-being (more depressive symptoms, less life satisfaction) during the early phase of the COVID-19 pandemic compared to 2019 than those not providing family care.* (Hypothesis 1a).

However, the well-being of family caregivers might also not have changed—or even increased—during the pandemic, as contextual changes (induced by the pandemic) and containment measures were able to provide relief for them. First, short-time work and work-from-home options may have helped to alleviate role overload and strain resulting from conflicts between jobs and caregiving (Ehrlich et al. 2020; Ehrlich 2023). Second, especially during the first phase of the pandemic in the first half of 2020, the performance of (professional) caregivers was greatly appreciated in the public. The increased social recognition and the feeling of fulfilling a socially crucial task under adverse conditions may have translated into a heightened sense of identity, which in turn enhanced well-being (Gray and Pattaravanich 2020). Recchi et al. (2020) identified an improvement in well-being in the general population in France during the early phase of the pandemic and speculated that it was due to social comparisons with those who were worse off: people in need of intensive care or dying from COVID-19. Those social comparisons might have been even more relevant for family caregivers, who compared the situation of their care recipient living at home with the situation of persons in care homes where many COVID-19 outbreaks took place with high death tolls among residents (Kohl et al. 2021) and social contacts were widely cut-off (Räker et al. 2021). With these considerations, we formulate a second contrasting hypothesis: *Those who provided family care will have experienced no change, or an increase in well-being (depressive symptoms, life satisfaction) during the early phase of the COVID-19 pandemic compared to 2019* (Hypothesis 1b).

Therefore, we expect effect heterogeneity within the group of family caregivers, stemming from differences in the intensity of care provision and the dynamics over time. The effect of the pandemic and the containment measures might especially vary between those who had already been providing family care before the pandemic and those who took up family care responsibilities during the pandemic. Räker et al. (2021) note that the pre-existing coping resources of caregivers can help master the pandemic situation and maintain pre-care well-being levels. Therefore, the group of *continuing family caregivers* is more likely to have had strategies to handle the burden and stress from care provision that could also help them cope with the adverse conditions during the pandemic. Consequently, for continuing family caregivers, increased social recognition and a heightened sense of identity might be relevant. Furthermore, continuing caregivers might have already adapted their well-being levels and might not, therefore, have experienced a further decline (Lacey et al. 2019; Sacco et al. 2020). New family caregivers probably did not have such strategies (yet). This would have been especially severe in a situation with reduced or no support from out- and inpatient care services. Therefore, we expect that *the relationship between family care provision and well-being is moderated by the dynamics of family care provision; the decline in well-being (more depressive symptoms, less life satisfaction) will have been more severe for new family caregivers* (Hypothesis 2a), *while continuing family caregivers will have experienced no change, or even an increase in well-being* (Hypothesis 2b).

As described above, the greater the care intensity, i.e. the higher the number of care hours provided, the more negative the effect on the well-being of caregivers (Chen et al. 2019; Hirst 2005; Sacco et al. 2020; Verbakel et al. 2018). Again, transferring these results to the pandemic situation, and the partial or complete suspension of informal and/or formal support, the burden for those providing intensive care might have been even stronger, and the related loss in well-being even greater than in pre-pandemic times. Especially during the lockdown in 2020, support and care provided by neighbours and friends increased temporary (Ehrlich and Kelle 2022). This caregiving tasks—most probably provided due to geographical proximity—may have included sporadic help with household tasks or grocery shopping. Therefore, it is crucial to distinguish care intensity categories. Therefore, we assume that *the relationship between family care provision and well-being is moderated by care intensity, with a stronger decline in well-being (more depressive symptoms, less life satisfaction) the more hours of family care provided (high-intensity family caregiving)* (Hypothesis 3).

### Data and methods

We examined the impact of the COVID-19 pandemic on the well-being of family caregivers using data from the 2019 annual wave of the German Socio-Economic Panel (SOEP, Goebel et al. 2019) and the SOEP-CoV study. The SOEP-CoV sample is a sub-sample of the SOEP that was surveyed between 30 March and 28 June 2020. All SOEP households with a valid telephone number were contacted by telephone, and one adult person in the household was asked to participate in the survey. Half of the calls were made in the late afternoon or evening (51% in total) to ensure that the on-site working population could be reached. The analytic sample is balanced, only including respondents who participated in both the SOEP annual wave 2019 and SOEP-CoV 2020: a total of N = 6694 adult persons with a mean age of 53.1, and 51% women (weighted numbers). Complete information on all focal variables is available for 91% of our analytic sample. We used the multivariate imputation by chained equations (mice) algorithm by van Buuren and Groothuis-Oudshoorn (2011) to multiply imputed missing values. Online Appendix B presents further information on the nonresponse patterns.^1^

The two outcome variables are general life satisfaction and depressive symptoms. General life satisfaction is asked on an 11-point Likert scale (0 completely dissatisfied, …, 10 completely satisfied). Depressive symptoms are measured by the PHQ-4, a standard instrument for measuring depression and anxiety consisting of four items with a 4-point scale (1 never, …, 4 every day), added to a sum score (e.g. Löwe et al. 2010). The related question is: “In the last two weeks, how often have you been bothered by any of the following problems? (1) having a lack of interest or pleasure in your activities, (2) feeling down, depressed or hopeless, (3) feeling nervous, worried or on edge, (4) feeling unable to stop or control your worry.” The central explanatory variable in our study is family care provision. Respondents are asked in the annual SOEP waves and in the SOEP-CoV study: *“What is a typical day for you? How many hours do you spend on the following activities on a typical weekday: care and support for individuals in need of care?”*. In line with previous research (e.g. Hirst 2005; Sacco et al. 2020), we consider family care in three categories: 1) no family care (0 h per weekday), 2) low-intensity family care (between 1 and 2 h per weekday, i.e. up to 10 h of family care per week), 3) high-intensity family care (more than 2 h per weekday, i.e. more than 10 h of family care per week). Figure A1 in the Online Appendix includes the mean values of depressive symptoms scores and general life satisfaction in 2019 and 2020 along care categories (with 95% confidence intervals and for N = 6694 individuals). As covariates, we include changes in *employment status* (categories: employment, unemployment and non-employment, including retirement and housekeeping) and changes in *concerns about caregivers’ own financial situation* (categories: very concerned, somewhat concerned, not concerned at all). Thereby, we control changes in the employment or financial situation that might impact individuals’ well-being. Weighted sample statistics for the analytic (balanced) sample for 2019 and 2020 and for the analytic (balanced) sample 2018 and 2019 are given in Appendix Table A1 and split up along caregiver categories in Appendix Table A2. The proportion of missing values in each focus variable is given in Appendix Table A3.

To test our hypotheses and gain insights into the impact of changes in family care at the start of the pandemic in spring 2020 compared to 2019, we estimate first difference models with a balanced panel of 2019 and 2020. First difference models only analyse change (within effects) while controlling for group differences and unobserved heterogeneity on the individual level (Allison 2009).^2^ We proceed in three steps. First, we estimate separate models for non-caregivers, new family caregivers (who took on care responsibilities shortly before or during the onset of the pandemic), and continuing family caregivers (who had been providing care before the pandemic). Note that caregiving status is defined *stable over time* so that individuals do not change the classification between the two survey years. We model the general change between 2019 and 2020 with a period dummy for 2020 for non-caregivers, new caregivers and continuing caregivers. For *each of these groups* our models take the following form:

$$\begin{aligned} y_{it} = & a_{i} + \gamma_{1} T_{it} + \gamma_{2} EMPLOY_{it} + \gamma_{3} CONCERNS_{it} \\ & + \gamma_{4} T_{it} CARE_{i} + \gamma_{5} EMPLOY_{it} CARE_{i} + \gamma_{6} CONCERNS_{it} CARE_{i} + \varepsilon_{it} \\ \end{aligned}$$

Here, $y_{it}$ maps the outcome variable of person $i$ at time $t$, $a_{i}$ constitutes the individual specific intercept, $T_{it}$ the survey year, $EMPLOY_{it}$ is the employment situation of $i$ at $t$ and $CONCERNS_{it}$ maps the concerns about the (non-)caregiver’s financial situation at year $t$. $\varepsilon_{it}$ is the error term of the model which is normally distributed with mean zero and variance $\sigma^{2}$. $\gamma_{k}$ are the parameters to be estimated. $EMPLOY_{it}$ $CONCERNS_{it}$ $y_{it}$

Second, to test whether changes in depressive symptoms and life satisfaction scores are significantly different between non-caregivers, new and continuing family caregivers, we use fully interacted models with the three groups non-caregivers, continuing caregivers, new caregivers ($CARE_{i}$). The change scores between 2019 and 2020 in depressive symptoms and life satisfaction are then interacted by these categories. The related model equation is

$$\begin{aligned} y_{it} = & a_{i} + \gamma_{1} T_{it} + \gamma_{2} EMPLOY_{it} + \gamma_{3} CONCERNS_{it} \\ & + \gamma_{4} T_{it} CARE_{i} + \gamma_{5} EMPLOY_{it} CARE_{i} + \gamma_{6} CONCERNS_{it} CARE_{i} + \varepsilon_{it} \\ \end{aligned}$$

Third, we integrate care intensity in the analysis by categorising caregivers in six groups: continuing low-intensity caregivers, continuing high-intensity caregivers, new low-intensity caregivers, new high-intensity caregivers, continuing caregivers switching from high- to low-intensity, and continuing caregivers switching from low- to high-intensity care (see Appendix Table A4). Again, these groups are operationalized as stable over time. We then use fully interacted models including only caregivers categorised in these six groups ($CAREINT_{i}$). The change scores between 2019 and 2020 in depressive symptoms and life satisfaction were again interacted by these categories. The related model equation is

$$\begin{aligned} y_{it} = & a_{i} + \gamma_{1} T_{it} + \gamma_{2} EMPLOY_{it} + \gamma_{3} CONCERNS_{it} \\ & + \gamma_{4} T_{it} CAREINT_{i} + \gamma_{5} EMPLOY_{it} CAREINT_{i} + \gamma_{6} CONCERNS_{it} CAREINT_{i} + \varepsilon_{it} \\ \end{aligned}$$

Note that our approach differs from a Difference-in-differences (DiD) design as the Corona pandemic was experienced by all sample members, i.e. there is no “control group” in our design. As a robustness check, we also analysed the data from a balanced panel for 2018–2019 with the same statistical strategy as for the 2019–2020 data (see Online Appendix C). Of course, this does not eliminate the problem of the missing control group; therefore, differences we find between 2019 and 2020 might have other causes than the pandemic.

We used the statistical software R (version × 64 3.6.2); the packages ‘Hmisc’ (Harrell 2022), ‘weights’ (Pasek 2021) and ‘diagis’ (Helske 2021) for descriptive statistics, the package ‘mice’ (van Buuren et al. 2022a, b) for multiple imputation, and the package ‘plm’ for regression analysis (Croissant et al. 2022).^3^

## Results

### Differences according to caregiving dynamics

Before we turn to the first difference regression models, we examine the sample composition as presented in the weighted sample statistics in Appendix Table A2. These descriptive statistics show some compositional differences between the three groups of non-caregivers, new caregivers who started caregiving in 2020, and continuing caregivers who provided care in 2019 and 2020. Compared to non-caregivers, continuing caregivers concentrated more in the higher age group of 61 years and older, and new caregivers in the middle age group of 41–60 years. While new caregivers had a similar female share as non-caregivers, the share of women was higher among continuing caregivers (67% female as compared to 50% among non-caregivers). The share of non-employment was higher among continuing caregivers (44% as compared to 35% among non-caregivers in 2019) and the share of unemployment higher among new caregivers as compared to non-caregivers (10% as compared to 4% among non-caregivers). Financial concerns were higher among new caregivers. However, the amount of *change over time* in employment status and concerns about financial situation were similar in the three groups except for a higher rate of transition to non-employment, including retirement, among continuing caregivers.

Tables 1 and 2 show the results of first difference regression models for 2019 and 2020. We began by testing changes in depressive symptoms between 2019 and 2020 for non-caregivers, continuing caregivers and new caregivers. Did individuals providing family care during the early phase of the COVID-19 pandemic experience a larger change or a smaller change than those not providing family care, or no difference, compared to 2019 (Hypotheses 1a and 1b)? For individuals who did not provide family care, our analysis reveals a significant overall increase in the depressive symptoms score of 0.65 scale units in 2020 compared to 2019 (Table 1, Model 1). Those who started providing family care between 2019 and spring 2020 had a significantly increased depressive symptoms score of 0.74 scale units in 2020 (Table 1, Model 2), and those who were already providing family care before the pandemic had a significantly increased depression score of 0.46 scale units in 2020 (Table 1, Model 3). Between 2018 and 2019, in contrast, there was a significant decrease in depression score among non-caregivers and continuing caregivers (see Models 1–3 in Appendix Table C2).

**Table 1:** First difference regression results from separated Models 1–3 and combined fully-interacted Model 4 on depression scores (Betas), 2019–2020

	Model 1: non-caregivers	Model 2: new caregivers	Model 3: continuing caregivers	Model 4a: new versus non-caregivers	Model 4b: continuing versus non-caregivers	Model 4c: new versus continuing caregivers
*Year*						
2020 (Ref.: 2019)	0.65* [0.03]	0.74* [0.15]	0.46* [0.15]	0.09 [0.14]	− 0.19 [0.15]	0.28 [0.20]
*Employment (Ref.: employed)*						
Unemployed	0.14 [0.17]	− 0.87 [0.66]	− 0.67 [0.72]	− 1.01 [0.61]	− 0.81 [0.75]	− 0.20 [0.94]
non-employed	− 0.10 [0.11]	− 0.25 [0.45]	0.43 [0.62]	− 0.15 [0.42]	0.53 [0.63]	− 0.68 [0.74]
*Concerned about own econ. Situation (Ref.: not at all)*						
Somewhat	0.39* [0.06]	0.55* [0.27]	0.22 [0.27]	0.16 [0.25]	− 0.17 [0.28]	0.33 [0.36]
Very	1.19* [0.11]	2.00* [0.45]	1.69* [0.44]	0.81 [0.43]	0.50 [0.46]	0.31 [0.60]
R squared	0.08	0.11	0.08	0.08	0.08	0.08
Sample size (individuals)	5,838	359	291	6488	6488	6488

Standard errors from pooled results are given in brackets. N = 206 persons stopped caregiving from 2019 to 2020. Sample size is the mean of the group sizes after multiple imputation

*p < 0.05. First difference analysis from m = 20 multiply imputed data sets

**Table 2:** First difference regression results from separated Models 1–3 and combined fully-interacted Model 4 on life satisfaction (Betas), 2019 – 2020

	Model 1: non-caregivers	Model 2: new caregivers	Model 3: continuing caregivers	Model 4a: new versus non-caregivers	Model 4b: continuing versus non-caregivers	Model 4c new versus continuing caregivers
*Year*						
2020 (Ref.: 2019)	0.19* [0.02]	0.19* [0.10]	0.13 [0.11]	0.01 [0.10]	− 0.06 [0.11]	0.06 [0.15]
*Employment (Ref.: employed)*						
Unemployed	− 0.03 [0.12]	0.31 [0.45]	− 0.64 [0.51]	0.34 [0.45]	− 0.61 [0.53]	0.95 [0.67]
Non-employed	0.02 [0.08]	0.60* [0.29]	0.24 [0.43]	0.58 [0.29]	0.22 [0.44]	0.36 [0.52]
*Concerned about own econ. Situation (Ref.: not at all)*						
Somewhat	− 0.33* [0.04]	− 0.47* [0.19]	− 0.20 [0.20]	− 0.14 [0.18]	0.13 [0.20]	− 0.27 [0.27]
Very concerned	− 0.89* [0.07]	− 1.06* [0.30]	− 0.67* [0.32]	− 0.17 [0.29]	0.22 [0.44]	− 0.39 [0.43]
R squared	0.04	0.07	0.04	0.04	0.04	0.04
Sample size (individuals)	5838	359	291	6488	6488	6488

Standard errors from pooled results are given in brackets. N = 206 persons stopped caregiving from 2019 to 2020. Sample size is the mean of the group sizes after multiple imputation

*p < 0.05. First difference analysis from m = 20 multiply imputed data sets

Models 4a–c contrast the three groups in paired comparisons based on a fully interacted regression. This means we can directly test whether changes in depressive symptoms and life satisfaction are significantly different between non-caregivers, new and continuing caregivers by adding interactions of each variable in the model with the three groups. Differences in change scores between (non-)caregivers are non-significant, indicating that increases in depressive symptoms between all three groups were similar. This was different between 2018 and 2019, when new caregivers experienced a significant increase in depressive symptoms compared to the reference group of non-caregivers (coefficient of 0.23* in Model 4a Appendix Table C2).

For general life satisfaction, our analysis shows a significant increase of 0.19 scale units in spring 2020 compared to 2019 for those who did not provide family care (Table 2, Model 1). The results for new and continuing caregivers also show a slight increase in life satisfaction of 0.19 and 0.13 scale units respectively, albeit non-significant for continuing caregivers (Table 2, Models 2–3). This is contrary to the development between 2019 and 2018 with a significant decline in life satisfaction in all three groups (see Models 1–3 in Appendix Table C3). Differences in change scores between non-caregivers, continuing caregivers and new caregivers are again non-significant in 2019–20 (Table 2, Models 4a–c), same as for 2018–2019 (see Models 4a–c in Appendix Table C3).

### Differences according to care intensity

For the multivariate analyses on differences according to caregiving intensity, caregivers were categorised in six groups depending on changes or stability in intensity as described above: continuing low-intensity caregivers, continuing high-intensity caregivers, new low-intensity caregivers, new high-intensity caregivers, continuing caregivers switching from high- to low-intensity, and continuing caregivers switching from low- to high-intensity care (see Appendix Table A4).

**Fig. 1:**
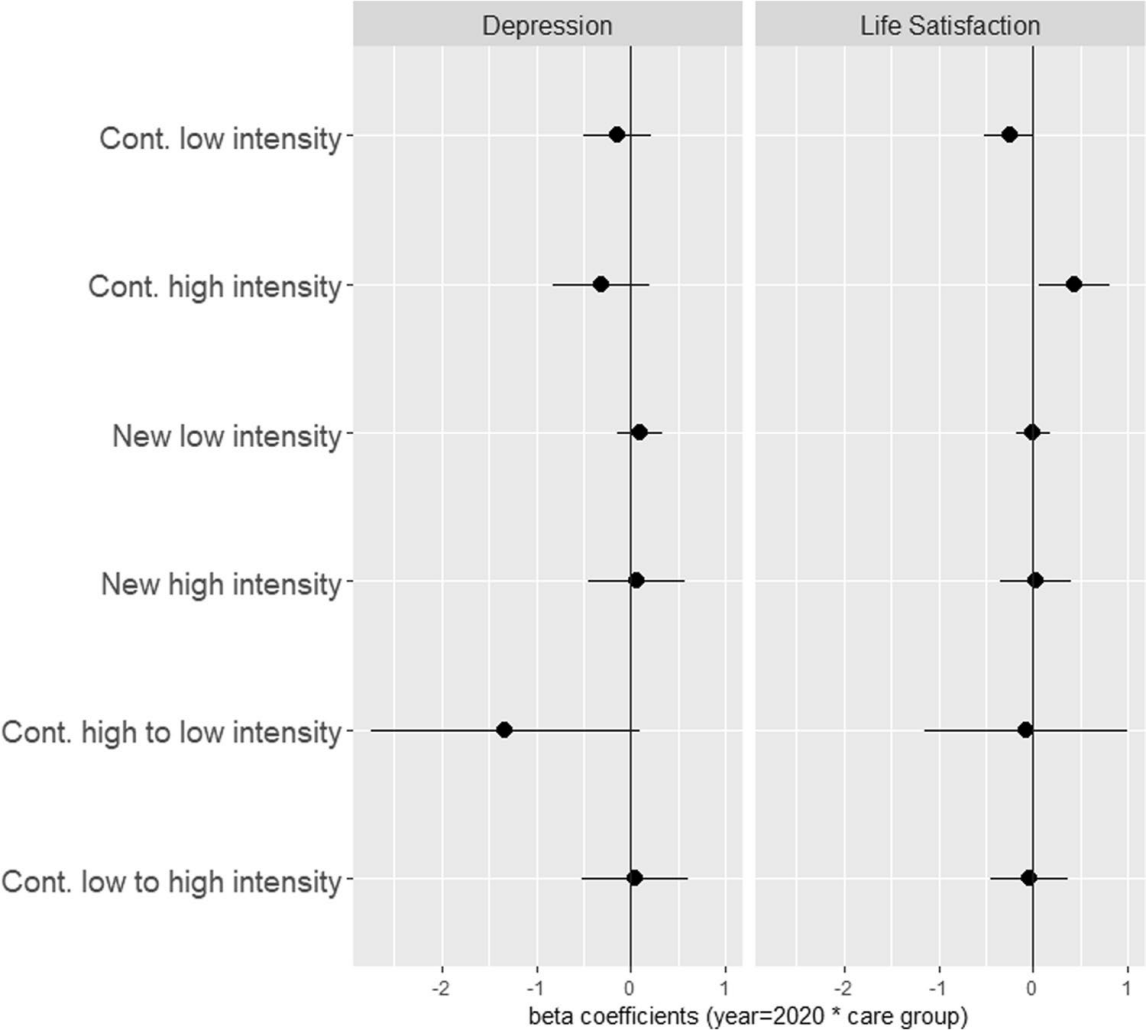
Coefficient plot for the first difference regression results on depression and life satisfaction (Betas), 2019–2020. *Notes*: Beta coefficients from regression models 1 and 2 in Appendix Table A5. Controlled for changes in employment (reference category: employed) and worries about financial situation (reference category: no worries)

Figure 1 shows selected beta coefficients from fully interacted models for the interactions of caregiving group*year 2020 (regression models are included in Appendix Table A5). These conditional effects show whether a change in well-being of a specific group of caregivers significantly deviated from non-caregivers. Note that the values apply with the reference categories set to ‘employed’ and ‘no financial worries’. The different types of caregivers did not significantly differ from non-caregivers in changes in depression scores (Fig. 1, left panel). Continuing low-intensity caregivers had a significantly smaller increase in life satisfaction than non-caregivers, while continuing high-intensity caregivers had a significantly larger increase in life satisfaction than non-caregivers (Fig. 1, right panel).

## Discussion

Viewing these results in light of our Hypotheses, we arrive at the following conclusions. Our conflicting Hypotheses 1a and 1b assuming a decline or increase in well-being of family caregivers between 2019 and 2020 compared to non-caregivers are neither confirmed nor refuted. Everybody, non-caregivers and caregivers alike, showed significant increases in life satisfaction and simultaneously (partly significant) increases in depressive symptoms. Increases in depression were larger for new caregivers who started family caregiving during or shortly before the first wave of the pandemic in 2020, and smaller for those who had continuing provided care already in 2019 and 2020, compared to non-caregivers. However, differences between the three groups are non-significant.

Our findings point to heterogeneity within the group of caregivers. While changes in well-being between 2019 and 2020 among caregivers are very much in line with changes in the general population, variation within the group of caregivers, especially due to care intensity, is striking—and partly contrary to our expectations. We assumed stronger negative effects from the pandemic for those providing high-intensity care (Hypothesis 3). In fact, increases in life satisfaction were largest for continuing high-intensity caregivers.

## Conclusion

Our study is the first, to the best of our knowledge, to examine relationships in the well-being of family caregivers in response to the first wave of the COVID-19 pandemic with detailed information on family care dynamics and intensity and appropriate data, sample and methods. The results on changes in depression scores and general life satisfaction of family caregivers over the course of the first COVID-19 lockdown show temporal and within-group differences regarding family care dynamics (new caregivers vs. continuing caregivers) and family care intensity (high- vs. low-intensity caregivers). While the changes in well-being between 2019 and 2020 among caregivers resemble those among non-caregivers, differences emerge in the group of continuing caregivers according to care intensity. Caregivers providing intensive care of more than two hours per day show a larger increase in life satisfaction than non-caregivers, while the opposite applies to low-intensity caregivers with two or less hours of care-provision per day. This difference might be due to the divergent care arrangements of these two groups. Continuing high-intensity caregivers are presumably primary caregivers or co-residential caregivers, as both caregiver types are associated with a higher number of average care hours (Ehrlich and Kelle 2019; Räker et al. 2020; Schneekloth et al. 2016). Low-intensity caregivers presumably receive help from a private or professional support network, are secondary caregivers, or are extra-residential caregivers – caregiver types that are all associated with a lower number of average care hours (Ehrlich 2018; Ehrlich and Kelle 2019; Schneekloth et al. 2016). Therefore, the latter group might be more negatively affected by containment measures, which greatly altered their care arrangements and restricted contact with persons outside their own household in the first phase of the COVID-19 pandemic. Furthermore, continuing high-intensity caregivers could be more resilient to the adverse conditions during the pandemic, having already become accustomed to the care situation before the onset of the pandemic in 2020. This group may have also benefited from the increased social reputation of care work during the first lockdown in the early phase of the pandemic, gaining increased self-esteem and a strengthened sense of identity—as predicted by role enhancement theory (Moen et al. 1995).

From a theoretical perspective, our study demonstrates that prominent theories on family care and well-being also have some predictive power during the COVID-19 outbreak and its subsequent containment measures. The results support the notion that family caregiving and the group of family caregivers are heterogeneous, presumably also due to differences in the ability to develop coping strategies (Haley and Pardo 1989; Seltzer and Li 2000; Townsend et al. 1989). The increase in life satisfaction among continuing high-intensity caregivers is in line with the role enhancement perspective. These caregivers may have felt a greater appreciation of their role as a family caregiver than before the pandemic. From a practical perspective, the situation of continuing low-intensity caregivers during the first wave of the pandemic, when professional care services were severely cut back, underlines the necessity of further expanding such options instead of cutting them, especially options that temporarily and flexibly reduce the care burden for family caregivers (Raiber et al. 2022; Stadler 2021).

However, our study has limitations. Generally, the association between family caregiving and the well-being of family caregivers might be endogenous; more resilient individuals might be more likely to take on care provision. Empirical research does not clearly support this “healthy caregiver hypothesis” (Fredman et al. 2010; Roth et al. 2015). Besides, the unexpectedness of the COVID-19 pandemic in early 2020 restricts the possibility for a selection effect in any direction; caregivers could not ‘opt-out’ of caregiving considering the difficulties ahead, and admission stops in care homes in 2020 further restricted opportunities to pass on care responsibilities to professional care services. Furthermore, with our first difference design, we focused on within variation, doing our best to account for unobserved heterogeneity, e.g. in terms of general health conditions and character traits. Unfortunately, we could not integrate information on the family caregiver’s support network, as this information is not available for all family caregivers. Moreover, the SOEP does not provide full information on when caring episodes began, so we could not integrate the exact duration of care episodes. Furthermore, due to this study’s small case numbers, gender-sensitive analyses were not applicable, although effects might be gendered (Swinkels et al. 2019; Zwar et al. 2020).

## Supplementary Information

Below is the link to the electronic supplementary material.

Supplementary file1 (DOCX 57 KB)Table A2 is in blue, not in black; please change

## Footnotes

1 A detailed description of the handling of nonresponse and imputation is given in the electronic supplementary material for this article, see Online Appendix B.

2 The first-difference estimator is obtained by a pooled ordinary least squares estimation for a regression of $y_{it} - y_{it - 1}$ on $x_{it} - x_{it - 1}$. Alternatively, our first difference approach may be denoted as change score approach.

3 The source code and further information on the data and methods is available at the GitHub link: https://github.com/bieneSchwarze/familyCareDuringCOVID19Lockdown
