# Supplementary material for: Family care during the first COVID-19 lockdown in Germany: longitudinal evidence on consequences for the well-being of caregivers
Source: Eur J Ageing. 2023 May 11;20(1):15. doi: 10.1007/s10433-023-00761-2 (PMC10173928; doi:10.1007/s10433-023-00761-2)
Supplement: Supplementary file 1 — Supplementary file1 (DOCX 57 KB)Table A2 is in blue, not in black; please change [file 10433_2023_761_MOESM1_ESM.docx]

# Online Appendix A: Tables and Figures

Manuscript: *Family care during the first COVID-19 lockdown in Germany: Longitudinal evidence on consequences for caregivers’ well-being*

**Figure A1**: Mean values of depression scores and general life satisfaction in 2019 and 2020 along care categories (with 95% confidence intervals and for N=6,694 persons).


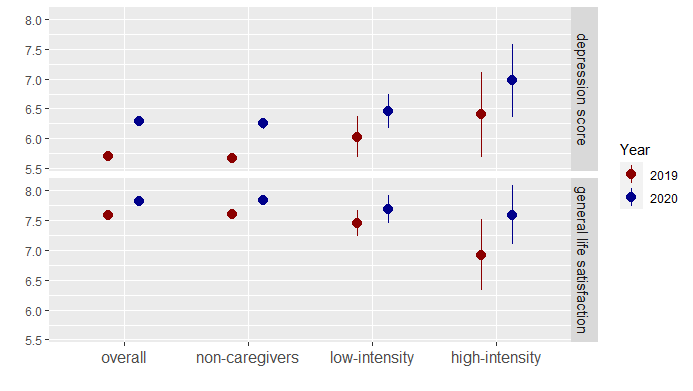


**Table A1**: Sample composition of the (balanced) panel samples for 2018-2019 and 2019-2020, column %.

|  | **Panel 2018-2019** | | **Panel 2019-2020** | |
| --- | --- | --- | --- | --- |
|  | **2018^†^** | **2019** | **2019** | **2020** |
| ***Age (in 2019)*** |  |  |  |  |
| 18-40 | 0.30 | | 0.30 | |
| 41-60 | 0.32 | | 0.32 | |
| 61 and older | 0.37 | | 0.38 | |
| ***Gender*** |  |  |  |  |
| male | 0.48 | | 0.49 | |
| female | 0.52 | | 0.51 | |
| ***Employment*** |  |  |  |  |
| employed | 0.60 | 0.60 | 0.60 | 0.59 |
| unemployed | 0.05 | 0.05 | 0.05 | 0.05 |
| non-employed | 0.35 | 0.35 | 0.35 | 0.36 |
| ***Concerned about fin. situation*** |  |  |  |  |
| very concerned | 0.10 | 0.12 | 0.11 | 0.09 |
| somewhat concerned | 0.44 | 0.45 | 0.43 | 0.39 |
| not at all concerned | 0.47 | 0.43 | 0.46 | 0.53 |
| ***Care Hours per day*** |  |  |  |  |
| non-caregiver | 0.94 | 0.94 | 0.93 | 0.91 |
| low-intensity caregiver | 0.05 | 0.05 | 0.05 | 0.06 |
| high-intensity caregiver | 0.01 | 0.02 | 0.02 | 0.03 |
| **Sample size** | 22,998 | | 6,694 | |

*Notes*: Weighted statistics. Some column cells do not sum up to 1 because of rounding **^†^**PHQ-4 was not asked in SOEP 2018; thus, we take the PHQ-4 measured in 2016 as a proxy.

**Table A2**: Sample composition of the (balanced) panel samples 2019-2020 along caregiver categories, column %.

|  | **Non-**  **Caregivers** | | **Continuous Caregivers** | | **New**  **Caregivers** | |
| --- | --- | --- | --- | --- | --- | --- |
|  | **2019** | **2020** | **2019** | **2020** | **2019** | **2020** |
| ***Age (in 2019)*** |  |  |  |  |  |  |
| 18-40 | 0.33 | | 0.13 | | 0.25 | |
| 41-60 | 0.31 | | 0.35 | | 0.46 | |
| 61 and older | 0.37 | | 0.52 | | 0.29 | |
| ***Gender*** |  |  |  |  |  |  |
| male | 0.50 | | 0.33 | | 0.49 | |
| female | 0.50 | | 0.67 | | 0.51 | |
| ***Employment*** |  |  |  |  |  |  |
| employed | 0.61 | 0.59 | 0.49 | 0.42 | 0.60 | 0.60 |
| unemployed | 0.04 | 0.05 | 0.07 | 0.07 | 0.10 | 0.09 |
| non-employed | 0.35 | 0.36 | 0.44 | 0.52 | 0.30 | 0.31 |
| ***Concerned about fin. situation*** |  |  |  |  |  |  |
| very | 0.11 | 0.08 | 0.13 | 0.09 | 0.16 | 0.16 |
| somewhat | 0.43 | 0.39 | 0.40 | 0.38 | 0.47 | 0.45 |
| not at all | 0.46 | 0.53 | 0.46 | 0.53 | 0.37 | 0.39 |
| **Sample size**^†^ | 5,557 | | 278 | | 339 | |

*Notes*: Weighted statistics. Some column cells do not sum up to 1 because of rounding. N=198 individuals gave up caregiving from 2019 to 2020 and for N=320 individuals care information for 2019 is missing.

**Table A3**: Proportion of missing values in each focal variable.

|  | **Panel 2018-2019** | | **Panel 2019-2020** | |
| --- | --- | --- | --- | --- |
|  | **2018^†^** | **2019** | **2019** | **2020** |
| Depression score | 0.27^‡^ | 0.01 | 0.04 | 0.01 |
| General life satisfaction | 0.07 | 0.00 | 0.04 | 0.02 |
| Employment | 0.06 | 0.00 | 0.04 | 0.00 |
| Concern own fin. situation | 0.06 | 0.01 | 0.04 | 0.01 |
| Informal care hours | 0.08 | 0.03 | 0.05 | 0.00 |
| **Sample size** | 22,998 | | 6,694 | |

*Notes*: **^†^**PHQ-4 was not asked in SOEP 2018; thus, we take the PHQ-4 measured in 2016 as a proxy. ^‡^Sample N and O not part of analysis set since these studies entered SOEP in 2018 and thus for them no depression measurement available in 2016 (used as proxy for 2018).

**Table A4**: Operationalisation of the combined care dynamics and care intensity variable

|  | Family care | | | | | | | |
| --- | --- | --- | --- | --- | --- | --- | --- | --- |
|  | 2019 | | | | 2020 | | | |
|  | No | Low | | High | No | Low | | High |
|  |  | | Intensity | |  | | Intensity | |
| Non-caregivers | 1 | 0 | | 0 | 1 | 0 | | 0 |
| Continuing low-intensity caregivers | 0 | 1 | | 0 | 0 | 1 | | 0 |
| Continuing high-intensity caregivers | 0 | 0 | | 1 | 0 | 0 | | 1 |
| Starting low-intensity caregivers | 1 | 0 | | 0 | 0 | 1 | | 0 |
| Starting high-intensity caregivers | 1 | 0 | | 0 | 0 | 0 | | 1 |
| Switching to low-intensity care | 0 | 0 | | 1 | 0 | 1 | | 0 |
| Switching to high-intensity care | 0 | 1 | | 0 | 0 | 0 | | 1 |

**Table A5:** Fixed effects regression results on depression and life satisfaction (Betas), 2019 – 2020

|  | | **Model 1: Depression** | **Model 2:  Life satisfaction** |
| --- | --- | --- | --- |
| **Year** (Ref.: 2019) | |  |  |
| 2020 | | 0.65* | 0.19* |
| **Year 2020 * Care group** (Ref.: non-caregivers) |  | | |
| Continuing low intensity caregivers | | -0.14 | -0.25* |
| Continuing high intensity caregivers | | 0.31 | 0.43* |
| New low intensity caregivers | | 0.10 | 0.00 |
| New high intensity caregivers | | 0.07 | 0.03 |
| Continuing caregivers switching from high to low intensity | | -1.33 | -0.07 |
| Continuing caregivers switching low to high intensity | | 0.05 | -0.04 |
| ***R squared*** | | 0.09 | 0.05 |
| ***Sample size (individuals)*** | | 6,488 | 6,488 |

*Notes*: * p<0.05. Fixed effects analysis from m=20 multiply imputed data sets. N=206 persons stopped caregiving from 2019 to 2020. Sample size is the mean of the group sizes after multiple imputation.
Controlling for changes in employment status and income worries.

# Online Appendix B: Nonresponse and imputation

In the related non-response analysis, particular attention was paid to employment status, income, gender, number of persons in a household, household type, educational level, migration background, and whether a person works in a systemically important occupation, as well as the Covid-19 incidence at NUTS-3 regional level (on the day of the interview). Post-stratification was based on distributions taken from the German Microcensus 2018 for various regional and socio-economic characteristics, including age, gender, household size, citizenship, size of municipality, and federal state. The derivation of the respective survey weights is described in Siegers et al. (2021).

The proportion of complete cases is less than 95%. Little’s (1988) test shows that the missingness mechanism is not missing completely at random. To counteract selection bias and at the same time increase the statistical power, we therefore multiply imputed missing values. For this purpose, we used the multivariate imputation by chained equations (mice) algorithm by van Buuren and Groothuis-Oudshoorn (2011), applying classification and regression trees (CART) as the imputation routine. To improve the predictive power of the imputation routine, we used several auxiliary variables in addition to the focal variables of this study (namely, migration background, educational attainment, household type, federal state). As suggested by Kim et al. (2006), we entered survey weights into the corresponding imputation models as explanatory variables. To maintain their autocorrelation and serial correlation structure, the data were imputed in wide format. We imputed m=20 data sets with 20 iteration steps in the Gibbs sampler of mice. We checked the convergence and meaningfulness of the estimated imputation models by means of the associated mice diagnostics (e.g., contrasting distributions of observed and imputed data).

**References**

van Buuren, S., & Groothuis-Oudshoorn, K., 2011. mice: multivariate imputation by chained equations in R. *Journal of Statistical Software*, 45(3), 1–67. https:// doi.org/10.18637/jss.v045.i03

Kim, J. K., Michael Brick, J., Fuller, W. A. & Kalton, G., 2006. On the bias of the multiple‑imputation variance estimator in survey sampling. *Journal of the Royal Statistical Society: Series B (Statistical Methodology)*, 68(3), 509-521.

Siegers, R., Steinhauer, H.W., & Zinn, S., 2021. Weighting the SOEP-CoV study 2020. SOEP Survey Papers 989888: SOEP Survey Papers: Series C - Data Documentations. https://www.diw.de/documents/publikationen/73/diw_01.c.820260.de/diw_ssp0989.pdf (accessed July 2021)

Little, R.J.A., 1988. A Test of Missing Completely at Random for Multivariate Data with Missing Values. *Journal of the American Statistical Association* 83, 1198–1202. https://doi.org/10.1080/01621459.1988.10478722

# Online Appendix C: Robustness analyses for 2018 and 2019

We executed robustness analyses for changes in depression scores and life satisfaction between 2018 and 2019. Non-caregivers and continuing caregivers experienced a significant decrease in depression scores (Table C2; Models 1 and 3). The change for new caregivers is non-significant (Table C2; Model 2), and the fully interacted model shows that new caregivers have a significantly lower decrease in depression score than non-caregivers (Table C2; Model 4a). All three groups showed a significant decrease in life satisfaction between 2018 and 2019 with no significant differences between the groups (Table C3).

The more detailed analyses by care intensity as presented in Table C4 reveal mostly no significant differences between the different groups of (non-)caregivers. Only new high intensity caregivers deviate significantly from non-caregivers with a lower decrease in depression score, while this does not apply to new low intensity caregivers.

**Table C1**: Mean values of general life satisfaction and depression score for 2018 and 2019.

|  | **2018^†^** | **2019** |
| --- | --- | --- |
| ***Depression score*** |  |  |
| overall | 6.18 (SD=2.23) | 5.86 (SD=2.34) |
| non-caregiver | 6.15 (SD=2.22) | 5.82 (SD=2.31) |
| low-intensity caregiver | 6.35 (SD=2.29) | 6.13 (SD=2.62) |
| high-intensity caregiver | 6.66 (SD=2.50) | 6.65 (SD=2.76) |
| ***General life satisfaction*** |  |  |
| overall | 7.68 (SD=1.62) | 7.50 (SD=1.70) |
| non-caregiver | 7.71 (SD=1.61) | 7.53 (SD=1.67) |
| low-intensity caregiver | 7.51 (SD=1.59) | 7.27 (SD=1.97) |
| high-intensity caregiver | 7.09 (SD=1.97) | 6.87 (SD=2.02) |
| **Sample Size** | 22,998 / 19,166^‡^ | |

*Note*: Weighted statistics. **^†^**PHQ-4 was not asked in SOEP 2018; thus, we take the PHQ-4 measured in 2016 as a proxy. ^‡^Sample N and O not part of analysis set since these studies entered SOEP in 2018 and thus for them no depression measurement available in 2016 (used as proxy for 2018). This reduces the sample size from 22,998 to 19,166.

**Table C2**: Fixed effects regression results from separated Models 1-3 and combined Models
4a-c on depression scores (Betas), 2018 – 2019

|  | **Model 1:**  **Non- Caregivers** | **Model 2:**  **New Caregivers** | **Model 3: Continuing  Caregivers** | **Model 4a:**  **New vs. Non-Caregivers** | **Model 4b:**  **Continuing vs. Non-Caregivers** | **Model 4c:**  **New vs.**  **Continuing Caregivers** |
| --- | --- | --- | --- | --- | --- | --- |
| **Year** |  |  |  |  |  |  |
| 2019 (Ref.: 2018) | -0.31* | -0.08 | -0.27* | 0.23* | 0.04 | 0.19 |
| **Employment** (Ref.: employed) | | | | | | |
| Unemployed | 0.29* | -0.08 | -0.39 | -0.37 | 0.10 | -0.47 |
| non-employed | 0.08 | 0.09 | 1.15* | 0.01 | 1.07* | -1.06* |
| **Concerned about own econ. Situation** (Ref.: not at all) | | | | | | |
| somewhat | 0.12* | 0.51* | 0.30 | 0.39 | 0.18 | 0.21 |
| very | 0.37* | 0.64 | 0.73 | 0.27 | 0.37 | -0.09 |
| ***R squared*** | 0.02 | 0.01 | 0.02 |  | 0.02 |  |
| ***Sample size (individuals)*** | 17,380 | 506 | 696 | 18,582 | 18,582 | 18,582 |

*Notes*: * p<0.05. Fixed effects analysis from m=20 multiply imputed data sets. Sample size is the mean of the group sizes after multiple imputation. ^‡^Sample N and O not part of analysis set since these studies entered SOEP in 2018 and thus no depression measurement available in 2016 (used as proxy for 2018). This reduces the sample size from 22,998 to 19,166. From these 19,166 persons, N=591 stopped caregiving from 2018 to 2019.

**Table C3:** Fixed effects regression results from separated Models 1-3 and combined Models
4a-c on life satisfaction (Betas), 2018 – 2019

|  | **Model 1:**  **Non- Caregivers** | **Model 2:**  **New Caregivers** | **Model 3: Continuing  Caregivers** | **Model 4a:**  **New vs. Non-Caregivers** | **Model 4b:**  **Continuing vs. Non-Caregivers** | **Model 4c**  **New vs.**  **Continuing Caregivers** |
| --- | --- | --- | --- | --- | --- | --- |
| **Year** |  |  |  |  |  |  |
| 2020 (Ref.: 2019) | -0.18* | -0.19* | -0.19* | -0.01 | -0.01 | 0.00 |
| **Employment** (Ref.: employed) | | | | | | |
| Unemployed | -0.17* | -0.19 | -0.26 | -0.02 | -0.09 | 0.07 |
| non-employed | 0.02 | 0.08 | -0.40 | 0.06 | -0.42 | 0.48 |
| **Concerned about own econ. Situation** (Ref.: not at all) | | | | | | |
| somewhat | -0.18* | -0.43* | -0.10 | -0.25 | 0.08 | -0.33* |
| very concerned | -0.50* | -0.63* | -0.79* | -0.13 | -0.29 | 0.16 |
| ***R squared*** | 0.02 | 0.04 | 0.02 |  | 0.03 |  |
| ***Sample size (individuals)*** | 20,867 | 606 | 827 | 22,299 | 22,299 | 22,299 |

*Notes*: * p<0.05. Fixed effects analysis from m=20 multiply imputed data sets. Sample size is the mean of the group sizes after multiple imputation. From N=22,998 persons in the data set, N=532 stopped giving care from 2018 to 2019.

**Table C4:** Fixed effects regression results on depression and life satisfaction (Betas), 2018 – 2019, separated by care intensity

|  | | **Model 1: Depression** | **Model 2:  Life satisfaction** |
| --- | --- | --- | --- |
| **Year** (Ref.: 2018) | |  |  |
| 2019 | | -0.31* | -0.18* |
| **Year 2019 * Care group** (Ref.: non-caregivers) |  | | |
| Continuing low intensity caregivers | | -0.04 | -0.07 |
| Continuing high intensity caregivers | | 0.11 | -0.07 |
| New low intensity caregivers | | 0.15 | 0.02 |
| New high intensity caregivers | | 0.53* | -0.17 |
| Continuing caregivers switching from high to low intensity | | 0.05 | 0.26 |
| Continuing caregivers switching low to high intensity | | 0.33 | 0.10 |
| ***R squared*** | | 0.02 | 0,03 |
| ***Sample size (individuals)*** | | 18,582 | 22,299 |

*Notes*: * p<0.05. Fixed effects analysis from m=20 multiply imputed data sets. Sample size is the mean of the group sizes after multiple imputation. Controlling for changes in employment status and income worries.
